# Anionic polycondensation and equilibrium driven monomer formation of cyclic aliphatic carbonates†

**DOI:** 10.1039/c8ra08219g

**Published:** 2018-11-20

**Authors:** Geng Hua, Peter Olsén, Johan Franzén, Karin Odelius

**Affiliations:** Department of Fibre and Polymer Technology, KTH Royal Institute of Technology SE-100 44 Stockholm Sweden hoem@kth.se +46-8-790-80-76; Department of Chemistry, KTH Royal Institute of Technology SE-100 44 Stockholm Sweden

## Abstract

The current work explores the sodium hydride mediated polycondensation of aliphatic diols with diethyl carbonate to produce both aliphatic polycarbonates and cyclic carbonate monomers. The lengths of the diol dictate the outcome of the reaction; for ethylene glycol and seven other 1,3-diols with a wide array of substitution patterns, the corresponding 5-membered and 6-membered cyclic carbonates were synthesized in excellent yield (70–90%) on a 100 gram scale. Diols with longer alkyl chains, under the same conditions, yielded polycarbonates with an *M*_w_ ranging from 5000 to 16 000. In all cases, the macromolecular architecture revealed that the formed polymer consisted purely of carbonate linkages, without decarboxylation as a side reaction. The synthetic design is completely solvent-free without any additional post purification steps and without the necessity of reactive ring-closing reagents. The results presented within provide a green and scalable approach to synthesize both cyclic carbonate monomers and polycarbonates with possible applications within the entire field of polymer technology.

## Abstract 2

One-pot two-step phosgene-free method to make functionalized 6-membered cyclic-carbonates and aliphatic polycarbonates in large quantity.

## Graphical abstract

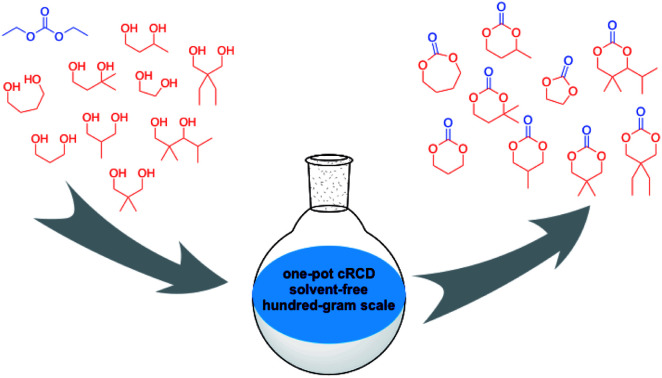

## Introduction

The production of valuable monomers through chemical recycling is considered central for a future sustainable society, as the methodology retains the material value and closes the loop for polymeric materials.^1–4^ If these processes are designed accordingly, multiple green chemistry principles including waste prevention, atom economy, and use of less hazardous chemicals can all be met at once.^5,6^ A prime example of this process can be found in the industrial production of lactide from poly(lactic acid).^7,8^

The ability of a polymer to reconvert back to its monomer relies on the thermodynamics of the reaction. Within this, the chemical structure of the repeating unit dictates both the feasibility and reaction conditions necessary to reconvert the polymer back to its monomeric form.^9–13^ The equilibrium behavior is clearly seen during the ring-opening polymerization of cyclic monomers, where, residual monomer will be present regardless of the reaction time and catalytic system employed.^14–17^ The implications of residual monomer in the material or in the reaction mixture depend on the end-use. Obviously, for polymer synthesis the amount of residual monomer should be as low as possible; however, for monomer synthesis the opposite is advantageous. The underlying reason for a system favoring or disfavoring polymerization is related to the thermodynamic features of the reaction such as the entropic increase in the system that drives monomer formation.^18,19^

Cyclic aliphatic carbonates constitute a class of monomeric building-blocks with applications covering the entire field of polymer science and also many more.^20–22^ Depending on the carbonate's ring-size, very different features for polymerization are obtained. For instance, most 5-membred cyclic carbonates are inert towards ring-opening polymerization at conventional conditions^23–25^ and are instead often used together with amines during isocyanate-free polyurethane synthesis. This is in sharp contrast to most of the 6-membered and larger cyclic carbonates, which propagate quickly under standard conditions and find applications ranging from refined biomedical polymers to bulk materials.^20,26,27^ The diversity in chemical structure of the cyclic carbonates relates to the well-developed and accessible ring-closing methodologies for an abundance of diols that can carry a wide range of functionalities and substituents. Classic ring-closing methodologies include, phosgene and triphosgenes,^28–30^ CDI (1,1′-carbonyldiimidazole),^31–33^ ethyl chloroformate,^26,34–37^ enzymes^38^ and many more.^39,40^ The common nominator for all these ring-closing systems is that the entropic increase is achieved *via* dilution to drive the reaction towards ring-closure.

Generally, during ring-opening polymerization there is a negative change in entropy, which means that ring-closing reactions may be induced by increasing the temperature, a reaction often referred to as ring-closing depolymerization (RCD). This concept is not new and was actually one of the first synthetic methodologies employed for the synthesis of these cyclic carbonates.^41^ Inherently, RCD has several advantages such as being solvent free, using non-toxic ring-closing reagents, being scalable and inexpensive (obviously depending on the diol), thus aligning well with the pursuit of sustainable chemistry. Nevertheless, reports on using this methodology for the synthesis of propagating cyclic carbonate monomers are comparably scarce, and the reaction conditions employed lacks coherency making general conclusions on the robustness and thermodynamics of RCD difficult.^41–46^ To shine some light on this methodology we intend to evaluate a wide range of different diols under the same reaction conditions to evaluate what system features that lead either to polycondensation or ring-closing depolymerization. We aim to highlight ring-closing depolymerization as the “go-to” method for cyclic carbonate monomer synthesis in a sustainable, large-scale and inexpensive manner. The vision is that this will highlight the importance of cyclic aliphatic carbonates in a future society based on renewable resources.

## Experimental

Sodium hydride (NaH, 60% dispersion in mineral oil), 2-methyl-1,3-propanediol (99%), 2,2-dimethyl-1,3-propanediol (99%), 1,3-propanediol (99%), 2,2-diethyl-1,3-propanediol (99%), 2,2,4-trimethyl-1,3-pentanediol (97%), (±)-1,3-butanediol (99.5%), 3-methyl-1,3-butanediol (97%), ethylene glycol (anhydrous, 99.8%) and 1,4-butanediol (99%) were purchased from Sigma-Aldrich. Diethyl carbonate (DEC, 99%) was purchased from Alfa Aesar. All chemicals were used as received.

### General oligomerization and controlled ring-closing depolymerization procedure

The depolymerization method and setup has previously been reported.^47,48^ For a typical depolymerization, 80 g of DEC (0.67 mol, 1.4 eq.) was charged into a 250 mL round bottom flask equipped with a magnetic stirring bar. 1 g of NaH (5% mol to hydroxyl groups) was added under nitrogen at room temperature. After a fine dispersion of NaH in DEC was formed, 37 g of 1,3-propanediol (0.48 mol, 1 eq.) was gradually added and once the slurry mixture turned colorless and transparent, the remaining diol was added. The temperature of the reaction vessel was then elevated to 120 °C and it was equipped with a distillation step up. Subsequently the condensate ethanol was distilled off overnight. The reaction vessel was cooled to 60 °C and vacuum was applied to further remove the ethanol and unreacted DEC. The temperature was then gradually elevated from 60 °C to 140–200 °C.

### Nuclear magnetic resonance (NMR)

The ^1^H-NMR (400.13 MHz) spectra were obtained from an Avance 400 (Bruker, USA) spectrometer at room temperature using CDCl_3_ or DMSO-d_6_ as solvent.

### Size exclusion chromatography (SEC)

The number average molar mass (*M*_n_) and dispersity (*Đ*) of the acetic acid quenched oligomers prior to RCD were analyzed with a Verotech PL-GPC 50 Plus system, equipped with a PL-RI detector and two Mixed-D (300 × 7.5 mm) columns (Varian, Santa Clara). An injection rate of 1 mL min^−1^ at 30 °C was used with chloroform as the mobile phase. Toluene was used as the internal standard for flow rate fluctuation corrections. Polystyrene standards with a narrow mass distribution and a molecular weight of 160–371 000 g mol^−1^ were used for calibration.

The product from the condensation reaction between the longer diols and DEC was analyzed with a TOSOH EcoSEC HLC-8320GPC system equipped with a RI detector, and two PSS columns (100 and 300), using *N*,*N*-dimethylformamide (DMF) with 0.01 M LiBr as the eluent. The analysis was conducted at 50 °C with a flow rate of 0.2 mL min^−1^. The results were plotted against linear PMMA standards.

## Results and discussions

**Fig. 1:**
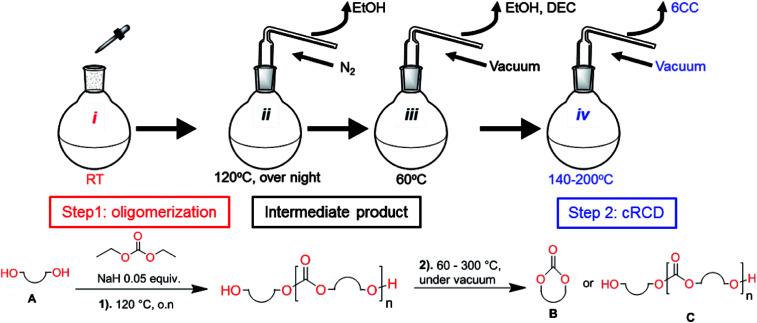
Synthetic methodology for the one-pot two-step oligomerization ring-closing depolymerization.

Aliphatic polycarbonates, either achieved *via* polycondensation or ring-opening polymerization of cyclic carbonate monomers, has the potential to be a key material class in a closed loop future sustainable society. We have previously shown that a one-pot and two-step reaction set-up illustrated in Fig. 1 is a feasible methodology for the synthesis a six-membered functional cyclic carbonate monomer, 2-allyloxymethyl-2-ethyltrimethylene carbonate (AOMEC).^46,49^ The first step comprises a slow addition of the diol to a suspension of sodium hydride in diethyl carbonate (DEC). The mixture was heated to 120 °C overnight to facilitate the condensation reaction between DEC and the alkoxide originating from the free diols or formed oligomeric species. After the formation of oligomers (Fig. 1, (i) and (ii)) the reaction vessel was cooled to 60 °C, vacuum was applied and the reaction vessel was subsequently reheated and the cyclic carbonate was collected as a distillate (Fig. 1, (iii) and (iv)). This provides us a very powerful methodology to synthesize a large amount of monomer in a short time frame. Inspired by this, we decided to explore the generality of this methodology both for the condensation reaction and also for the synthesis of a series of cyclic carbonates from diols with structural diversity.

### Equilibrium between condensation and depolymerization as a function of the diol length

To make aliphatic polycarbonates using diols and dialkyl carbonates as the starting materials, there are two ways to go: direct condensation or cyclization followed by ring-opening polymerization. Depending on the actual structure of the diol and the targeted polymer architecture, the strategy can be either route. In the condensation route, as exemplified in Fig. 1, polycarbonate is obtained as the desired product after step (i). The only tuning of the polymeric structure is from the change of the diol. In the cyclic route, the cyclic monomer is obtained after going through all four steps (i–iv). The polycarbonate is then obtained after a further step of ring-opening polymerization. The advantage of undergoing cyclization followed by ring-opening polymerization is the versatility of the ring-opening polymerization, which can result in a broad range of polymeric materials of varying chemical structures and architectures. It is well known that during ring-opening polymerization the ring size of the cyclic monomer and the temperature of the system play very important roles on the equilibrium between monomer and polymer. We therefore first focused on how the size of the unsubstituted α,ω-diols (Table 1) influenced the equilibrium between the cyclic monomer and polymer.

**Table 1:** Condensation behavior as a function of diol-length

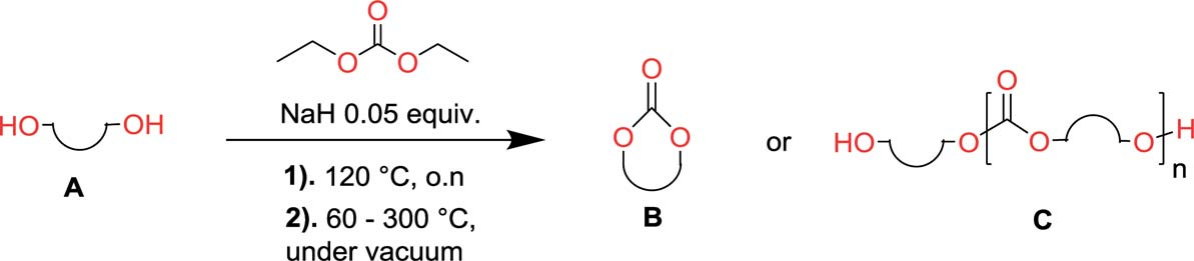							
Entry	*T*_max._ (°C)	Diol	Cyclic carbonate	Yield^a^ (%)	Oligomer	*M*_n_ (SEC)^b^	*Đ*
1	160	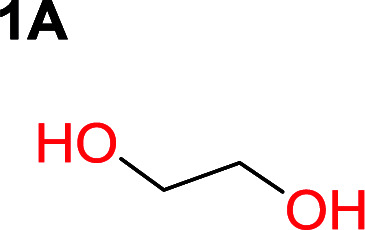	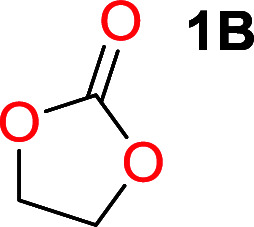	83	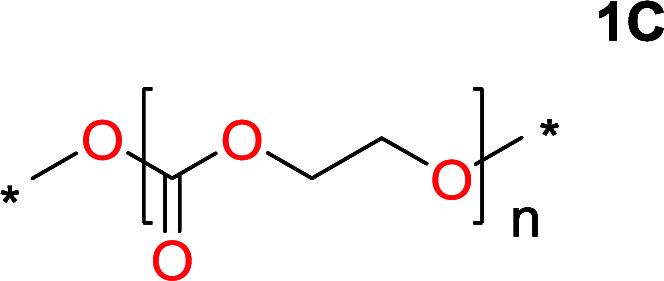	Not detected	—
2	210	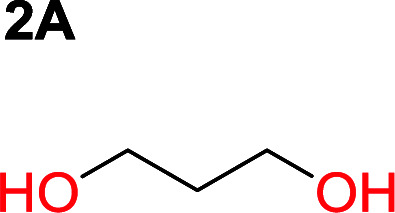	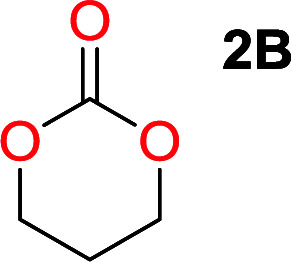	70	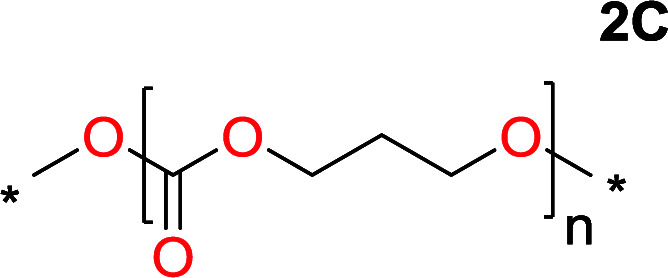	1900	2.7
3	260	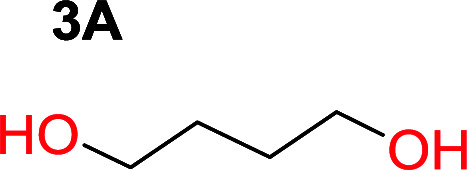	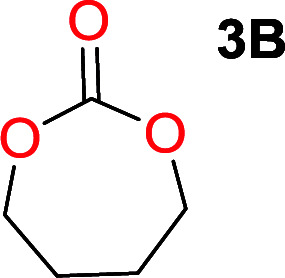	2	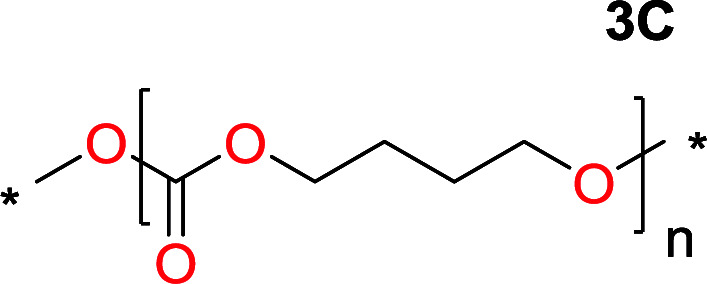	3500	2.0
4	260	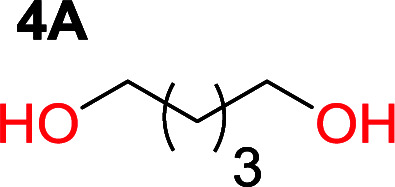	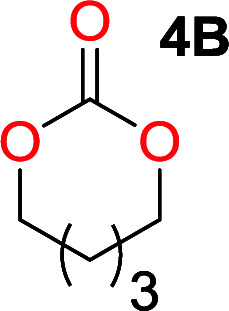	0	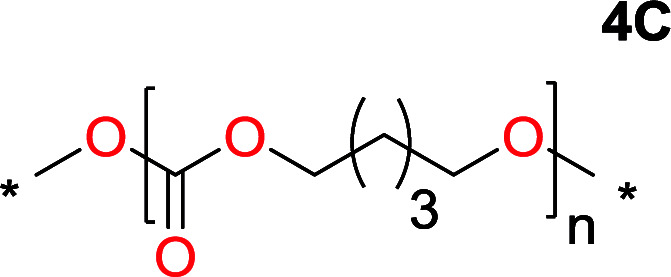	6200	2.6
5	260	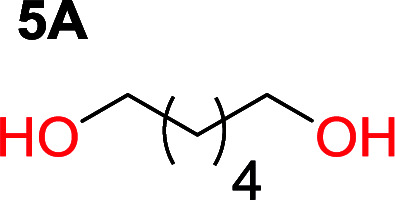	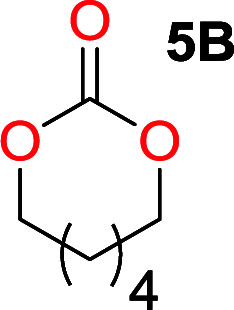	0	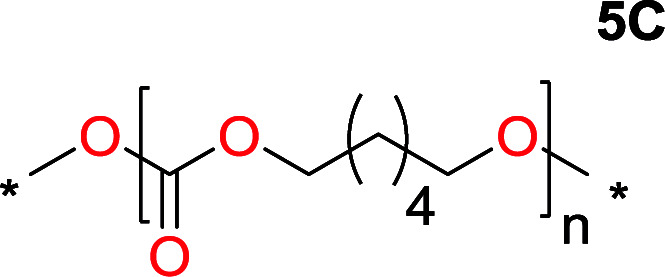	5200	1.8
6	260	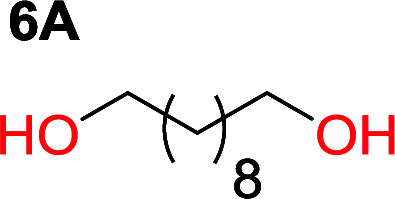	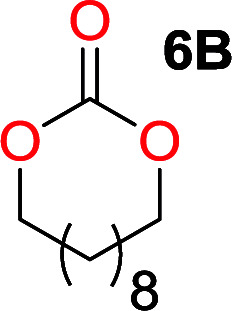	0	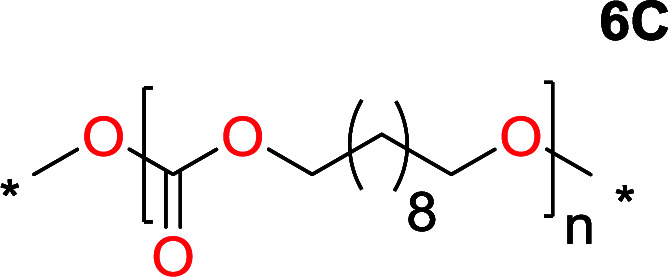	7000	1.1^c^

a Isolated yield.

b Determined from polystyrene standards in DMF.

c Problems with solubilizing the polymer in DMF.

It was obvious that both ethylene glycol and 1,3-propanediol formed 5- and 6-membered cyclic carbonates at high yields, 83% and 70% respectively (Table 1 entries 1 and 2). In our set-up, all other diols only formed the polycondensation product of the respected polycarbonates (Table 1 entries 3 to 6). The carbonate moiety within the polymeric structure was confirmed by ^13^C NMR (Fig. S1†). The inability of this methodology to ring-close larger carbonate rings is in contrast to what has previously been shown where larger cyclic carbonates may undergo ring-closing depolymerization at elevated temperatures.^41,44,50,51^ The features of this system is believed to be a consequence of the anionic environment produced *via* sodium alkoxide. Hence, under these reaction conditions, the diol-length fully controls if the reaction leads to either monomer or polymer formation.

### Substitution pattern and equilibrium tendencies

The influence from the diol-length on the formation of cyclic carbonate monomers and polycarbonates highlighted the beneficial equilibrium tendencies of ethylene glycol and 1,3-propane diol to form cycles compared to all other diols. Yet, from a polymer perspective, the six-membered carbonate ring is much more valuable than cyclic carbonates of other ring-sizes as it is known to be highly propagating. As an example, trimethylene carbonate (TMC), an unsubstituted six-membered monomer, is utilized particularly in biomedical applications as an important comonomer to l-lactide.^47,52,53^ The ring-closing depolymerization methodology was further expanded to six additional 1,3-diols with very different substitution patterns (Table 2). To our delight, all cyclic monomers were synthesized in high yields ranging from 70–90% (Table 2 entries 1–7). Most of the monomers required temperatures above 180 °C under vacuum to be distilled from the reaction mixture (Table 2 entries 1–5), except for the more heavily substituted carbonate 4,4-dimethyl-1,3-dioxan-2-one (Table 2 compound 6B) and 4-isopropyl-5,5-dimethyl-1,3-dioxan-2-one (Table 2 compound 7B), where the former had an onset distillation temperature 140 °C lower.

**Table 2:** Ring-closing depolymerization to 6-membered cyclic carbonates, substitution effect

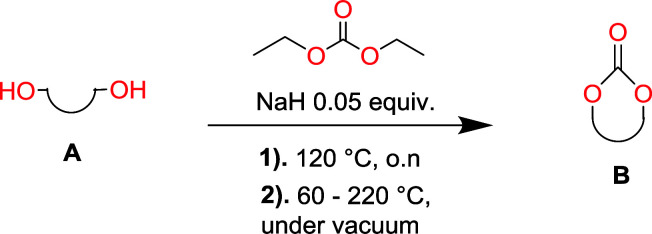					
Entry	Diol	Cyclic carbonate	Conv. to B after (1) (%)	*T*_distill._ (°C)	Yield^a^ (%)
1	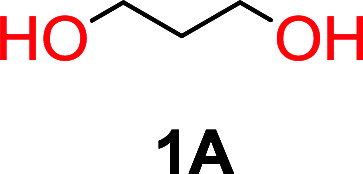	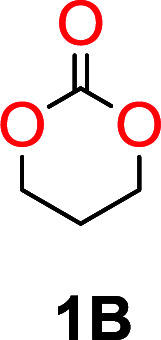	5	195–210	70
2	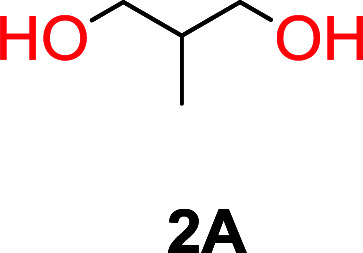	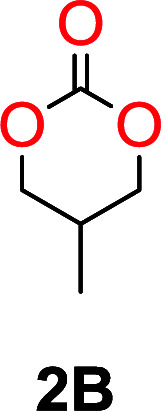	7	185–200	78
3	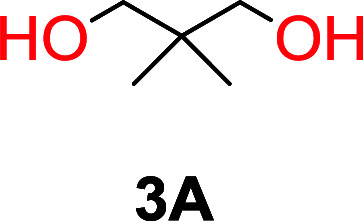	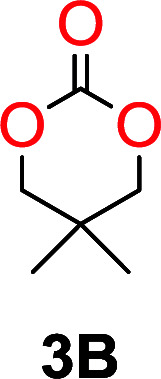	9	180–200	82
4	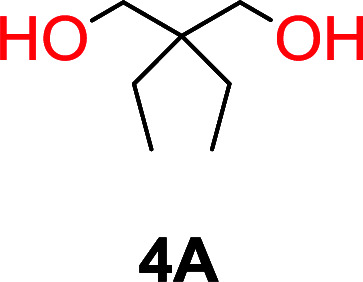	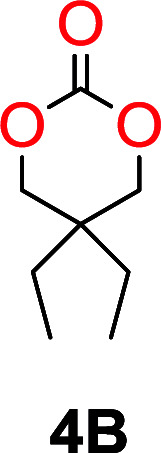	29	180–200	84
5	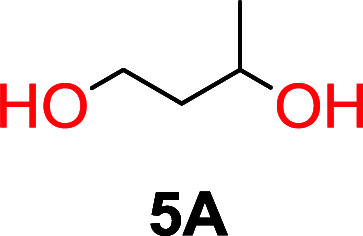	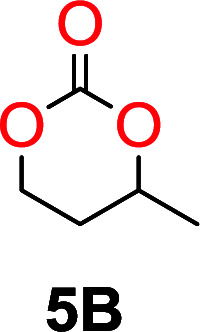	12	195–210	68
6	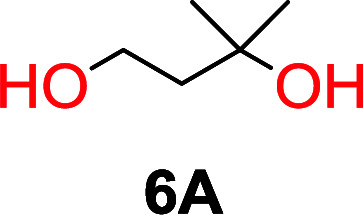	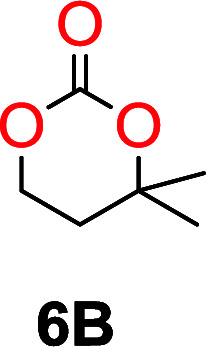	42	140–145	91
7	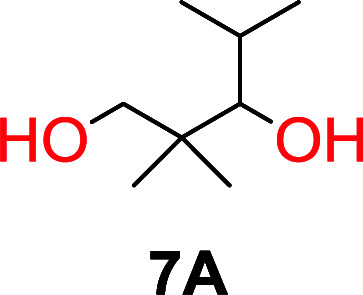	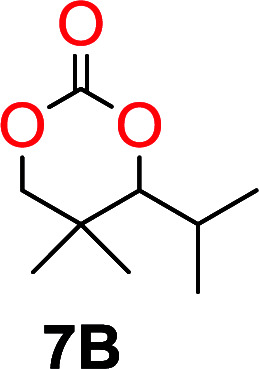	43	165–185	79

a Isolated yield.

**Fig. 2:**
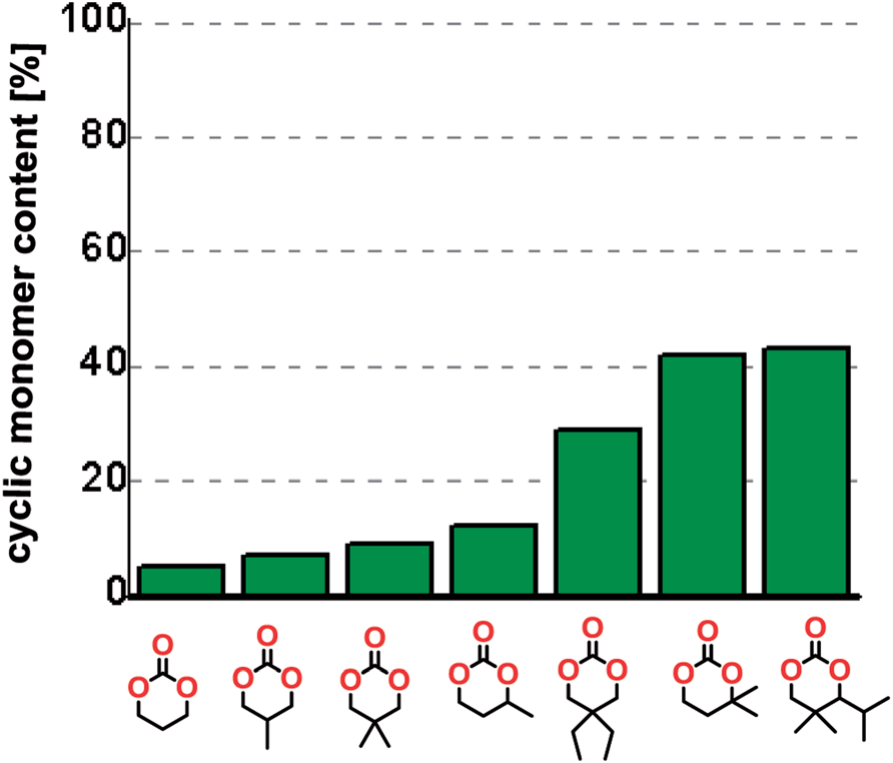
Ratio of monomer in raw product after the initial condensation step.

To understand the differences in equilibrium behavior of the monomers, the reaction composition after the initial condensation step (Fig. 1, (ii)) was analyzed through ^1^H NMR (Fig. S2–S9†). This provides a direct way to evaluate how the substitution pattern of the 1,3-propane diol relates to the equilibrium behavior of the monomer to oligomer (Fig. 2). It is well established that addition of substituents leads to an increased tendency towards ring-closure, more commonly referred to as the Thorpe–Ingold effect, and our system is in no way any different.^54,55^ Substitution on the 2-position of the cyclic carbonate influences the equilibrium behavior less as compared to the 1-position. Specifically, when comparing the unsubstituted TMC to the cyclic carbonate, that is mono-methylated on the 2-position, only an additional 2% of monomer in equilibrium is observed (Table 2, entries 1, 2 and Fig. 2). This in contrast to substitution on the 1-position of the carbonate, were one methyl group changes the equilibrium with 7% more in favor of the formation of the cyclic monomer (Table 2, entries 1,5 and Fig. 2). Similar trends have been seen for both cyclic carbonates and cyclic esters where both size of substitution and position have a large impact on the equilibrium tendencies between monomer and oligomer.^11,15,56,57^ The small changes in equilibrium behavior may seem insignificant (Fig. 2), however, from a synthetic perspective this will have a large consequences on the polymerization behavior.^15,58^

The equilibrium between aliphatic polycarbonates and cyclic carbonate monomers is related to the ceiling temperature (*T*_c_) of the polymerization. The ceiling temperature is defined as the temperature at which zero conversion of the monomer to polymer occurs. To achieve polymers it is highly advantageous to have a high *T*_c_ system, however this means that a high temperature is required to achieve ring closing depolymerization and the reaction conditions may lead to other side-reactions. The results obtained from the condensation step makes it possible to formulate several generic polymer synthetic conclusions based on the equilibrium observations (Fig. 2). The equilibrium behavior of the cyclic carbonate that on the 2-position is mono-substituted as well as the di-methylated suggest high conversion at conventional polymerization condition.^59^ In the case of the more substituted cyclic carbonates monomers the equilibrium reside more towards the cyclic carbonate monomer, thus suggesting a weaker polymerization behavior (Fig. 2).

**Scheme 1:**
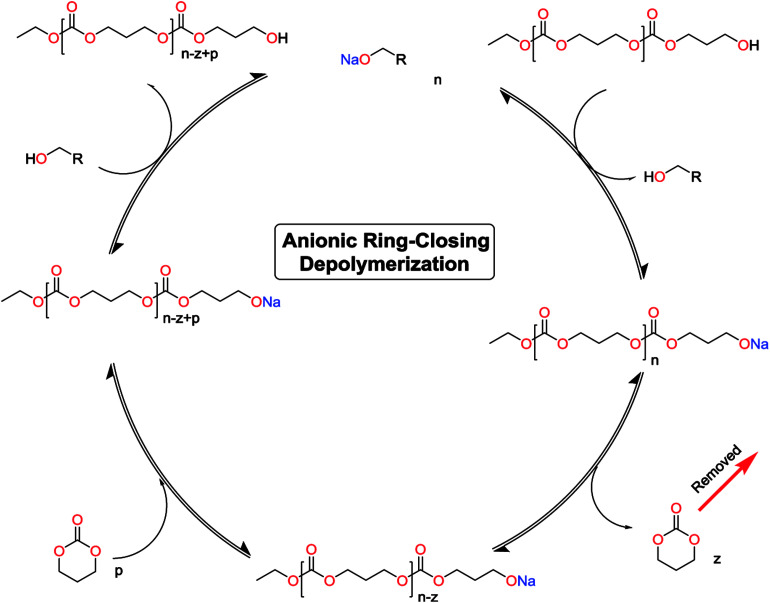
Proposed catalytic cycle for the anionic ring-closing depolymerization.

The monomer formation mechanism is proposed to occur through an anionic ring-closing depolymerization reaction sequence. The central component of this reaction is the anionic chain-end of the pre-formed oligomers that through a back-biting mechanism releases the cyclic monomer (Scheme 1). By constant removal of the cyclic monomer the reaction sequence can be driven towards high conversion. This is a very important difference compared to classic cyclization with ring-closing reagents, where the reaction outcome is determined by the initial features of the system. There are examples in the literature were larger carbonate monomers have been produced under depolymerization conditions, however our system did not result in these cycles (Table 1).^41,44,50,51^ This underlines the importance of the catalytic system to produce the desired outcome of the system. Recent report on the DBU and thiourea catalyzed shifts in equilibrium conversion points opens the possibility that the thermodynamic equilibrium is not completely independent of the catalytic system employed.^60^ This may very well be the key component for eventually expanding the scope of accessible monomers through ring-closing depolymerization methodologies.

## Conclusion

The length of the unsubstituted alkyl chain of the α,ω-diol controls the outcome of the reaction set-up by leading to either polycarbonates or cyclic carbonate monomers. Ethylene glycol and 1,3-propane diol produced the cyclic carbonates at high yields, 83% and 70% respectively. Substitution on the 2-position of the cyclic carbonate have a smaller effect on the equilibrium behavior compared to the substitution on the 1-position. By comparing trimethylene carbonate to the mono-methylated version on the 2-position only an additional 2% of monomer in equilibrium is observed, however the same substituent on the 1-position of the carbonate leads to more than 7% more of cyclic monomer in equilibrium.

The proposed mechanism for depolymerization is *via* an anionic induced ring-closing depolymerization from the chain end that releases the cyclic monomer. By constantly removing the cyclic monomer the reaction sequence can be driven towards high conversion. In all, we have successfully produced in total seven cyclic carbonates on a 100 g scale using a one-pot set-up without solvent or toxic reagents. All chemicals involved are commercially available and inexpensive and the experimental set-up is readily available. The ease in synthesis, scale and low cost highlight the immense potential of this class of monomers in the entire field of polymer science.

## Conflicts of interest

The authors declare no competing financial interest.

## Supplementary Material

RA-008-C8RA08219G-s001
