# Supplementary material for: Anionic polycondensation and equilibrium driven monomer formation of cyclic aliphatic carbonates
Source: RSC Adv. 2018 Nov 20;8(68):39022–8. doi: 10.1039/c8ra08219g (PMC9090641; doi:10.1039/c8ra08219g)
Supplement: RA-008-C8RA08219G-s001 [file RA-008-C8RA08219G-s001.pdf]

Electronic Supplementary Material (ESI) for RSC Advances.
This journal is © The Royal Society of Chemistry 2018

Electronic supplementary information (ESI)

Anionic polycondensation and equilibrium driven monomer formation of cyclic aliphatic carbonates

Geng Hua,[a] Peter Olsén,[a] Johan Franzén[b] and Karin Odelius[a,*]

a. Department of Fibre and Polymer Technology, KTH Royal Institute of Technology, SE-100 44, Stockholm, Sweden

b. Department of Chemistry, KTH Royal Institute of Technology, SE-100 44, Stockholm, Sweden

*Corresponding author: K.O. hoem@kth.se, +46-8 790 80 76

This ESI contains 9 figures in 10 pages.

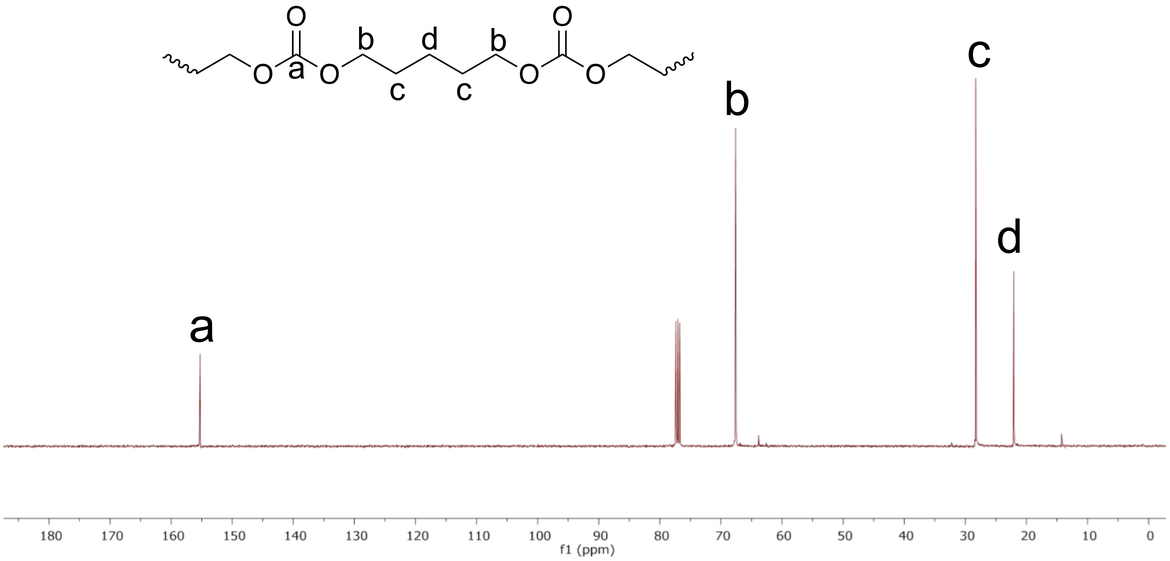

Figure S1 ^{13}C-NMR of the condensation product between 1,5-pentanediol and diethyl carbonate. The peak at 155.2 ppm represents the carbonate moiety in the condensation product.

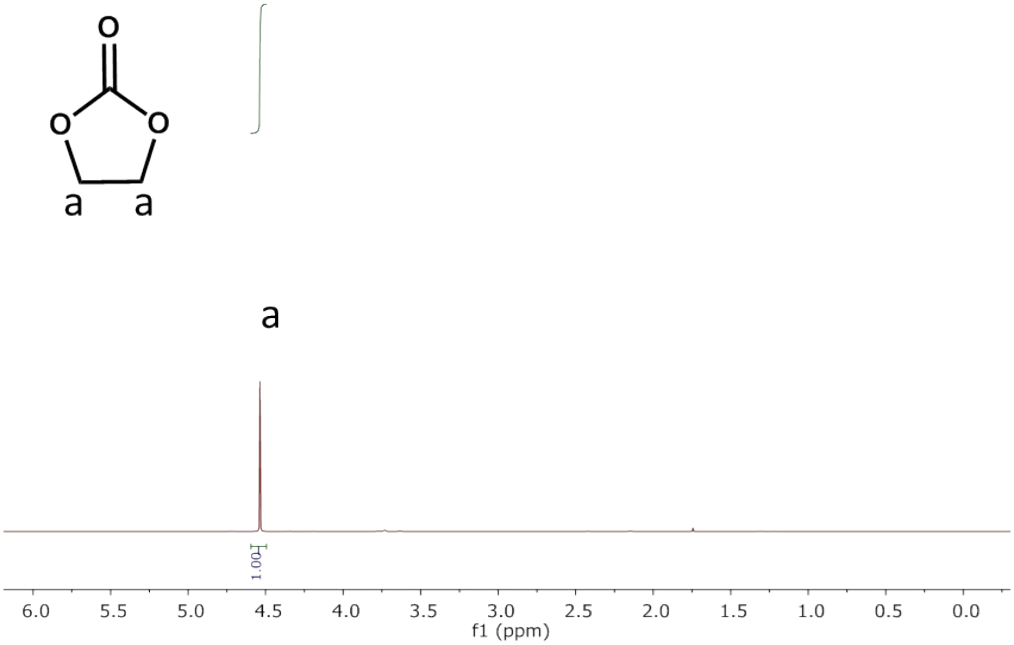

Figure S2 ^{1}H-NMR of 1,3-dioxolan-2-one. (5CC-1B)[1]

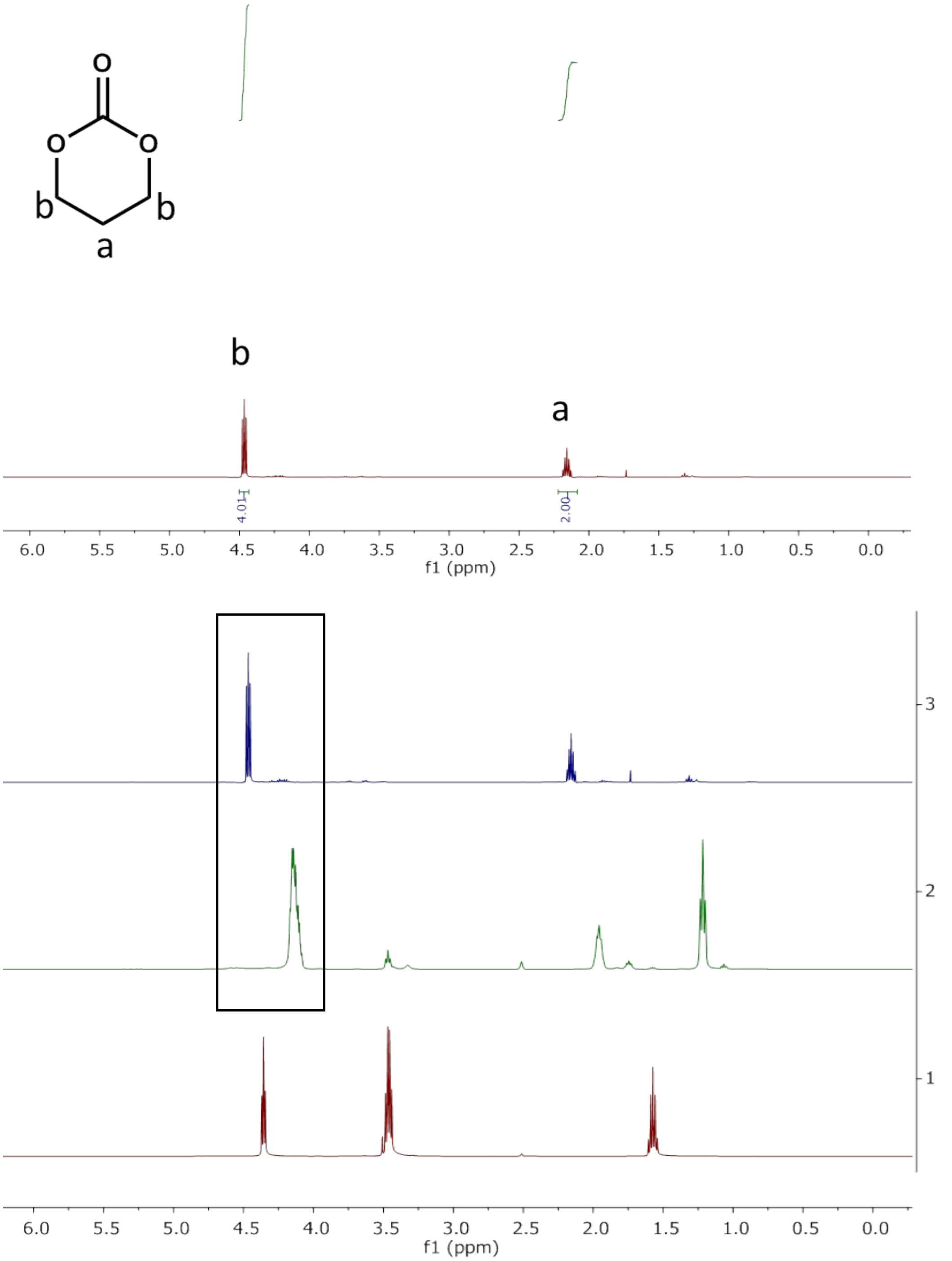

Figure S3 Upper, ^{1}H-NMR of 1,3-dioxan-2-one (6CC-1B).[2] Lower, ^{1}H-NMR compilation of 1) 1,3-propanediol, 2) oligomers prior to cRCD and 3) 1,3-dioxan-2-one.

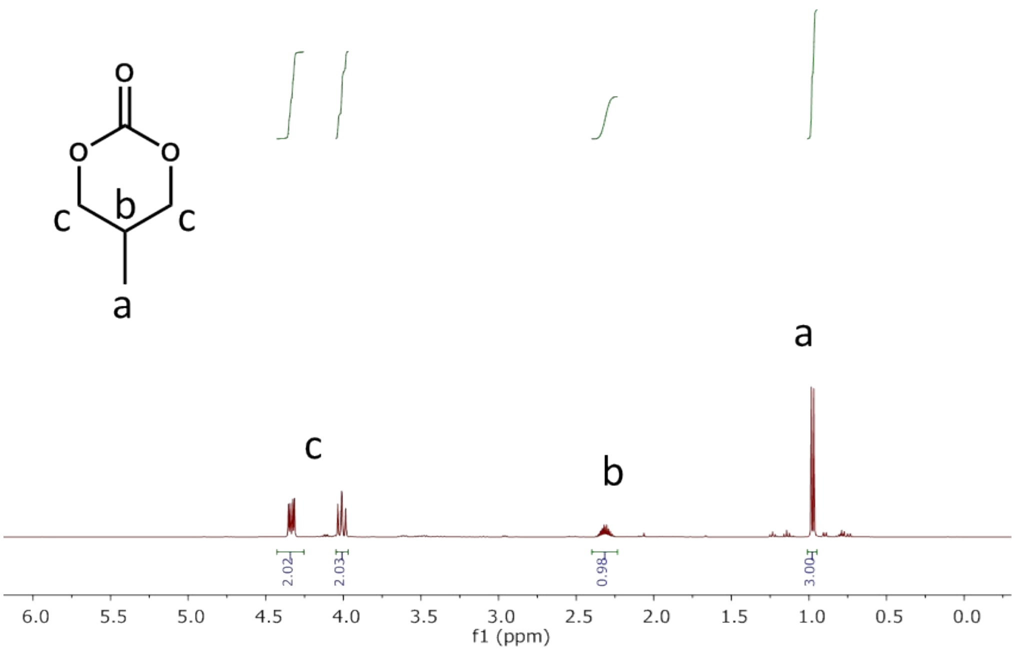

Figure S4 ^{1}H-NMR of 5-methyl-1,3-dioxan-2-one (6CC-2B).[3]

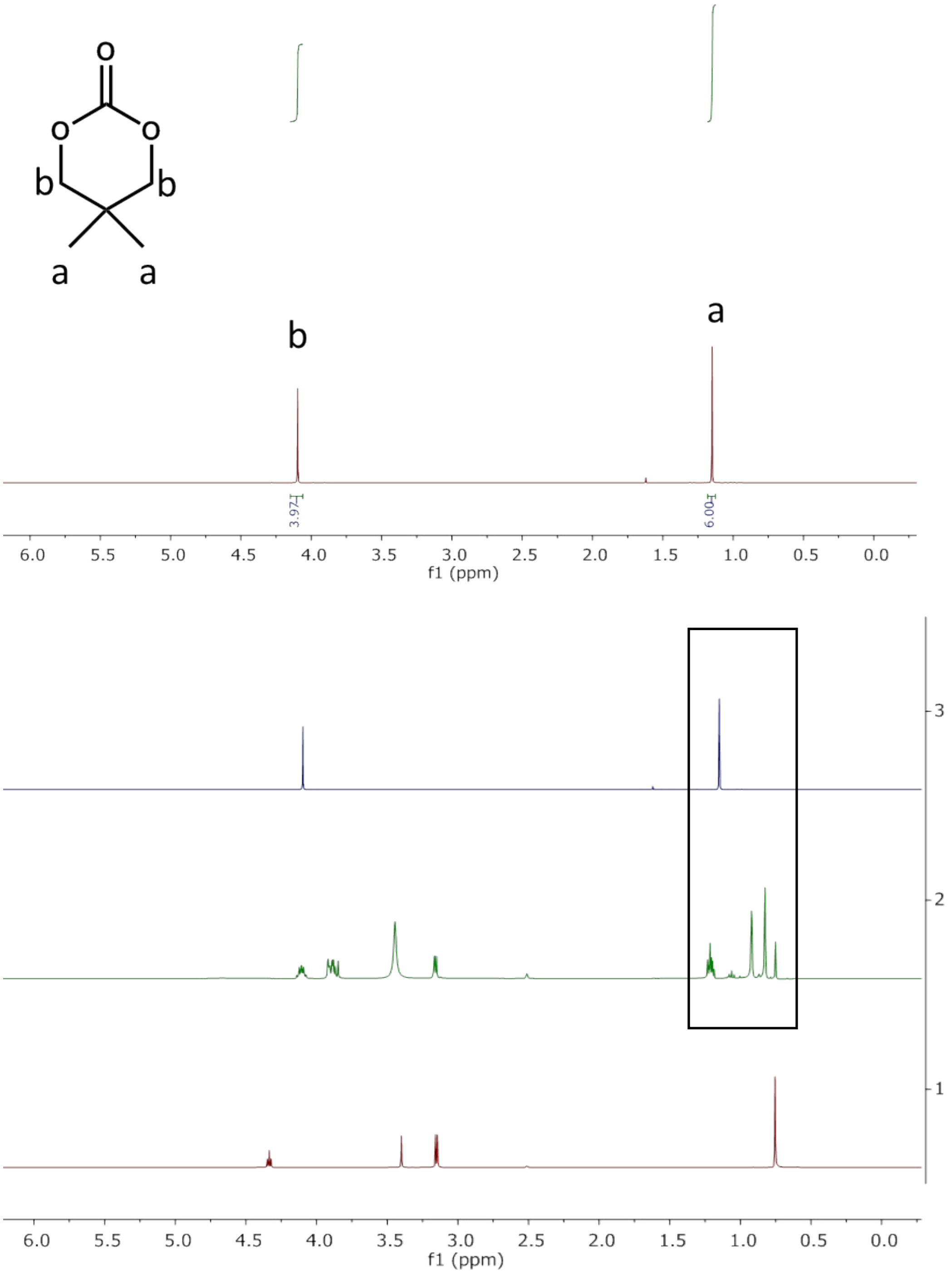

Figure S5 ^{1}H-NMR of 5,5-dimethyl-1,3-dioxan-2-one (6CC-3B).[2] Lower, ^{1}H-NMR compilation of 1) 2,2-dimethyl-1,3-propanediol, 2) oligomers prior to cRCD and 3) 5,5-dimethyl-1,3-dioxan-2-one.

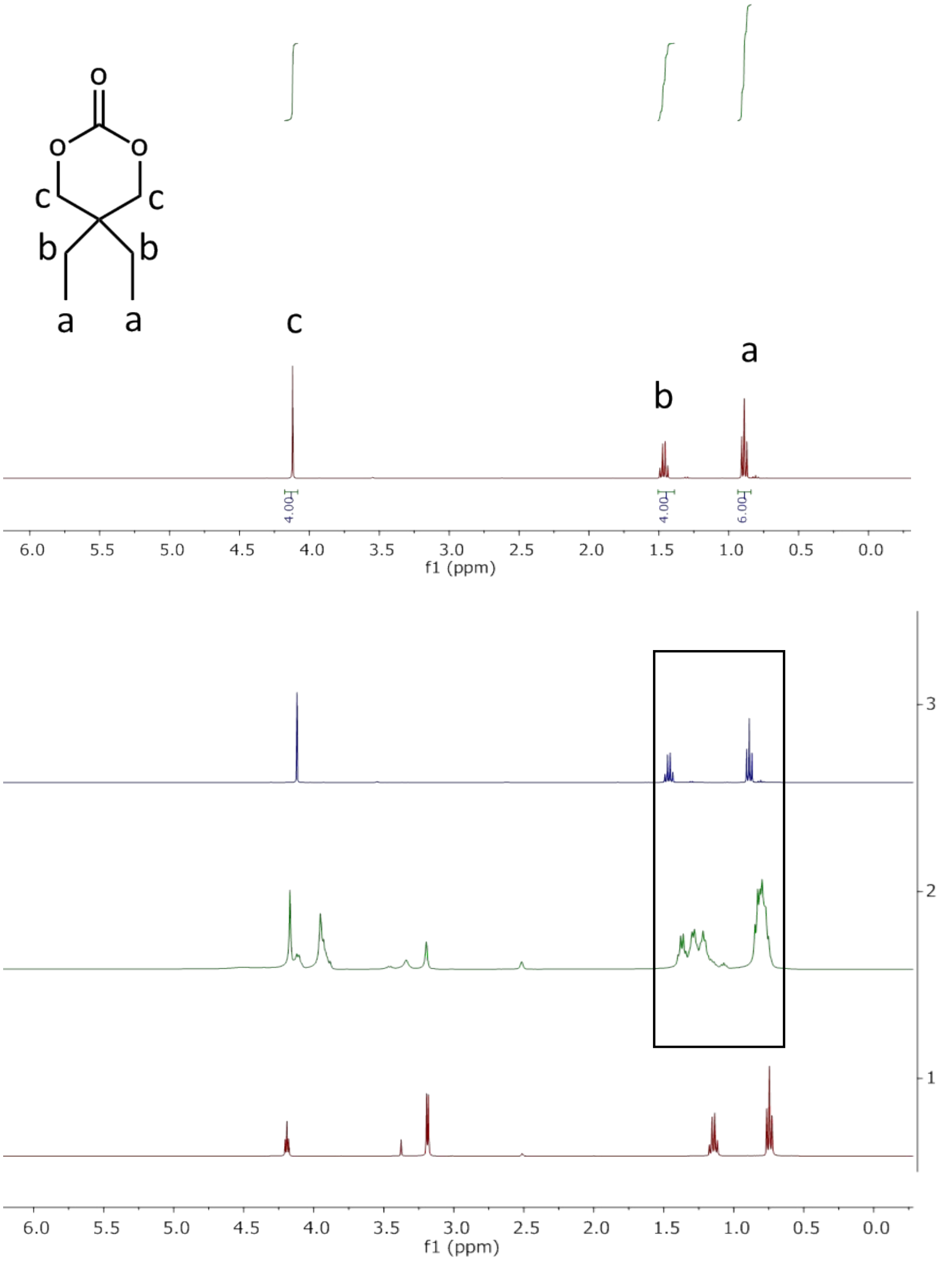

Figure S6 ^{1}H-NMR of 5,5-diethyl-1,3-dioxan-2-one (6CC-4B).[4] Lower, ^{1}H-NMR compilation of 1) 2,2-diethyl-1,3-propanediol, 2) oligomers prior to cRCD and 3) 5,5-diethyl-1,3-dioxan-2-one.

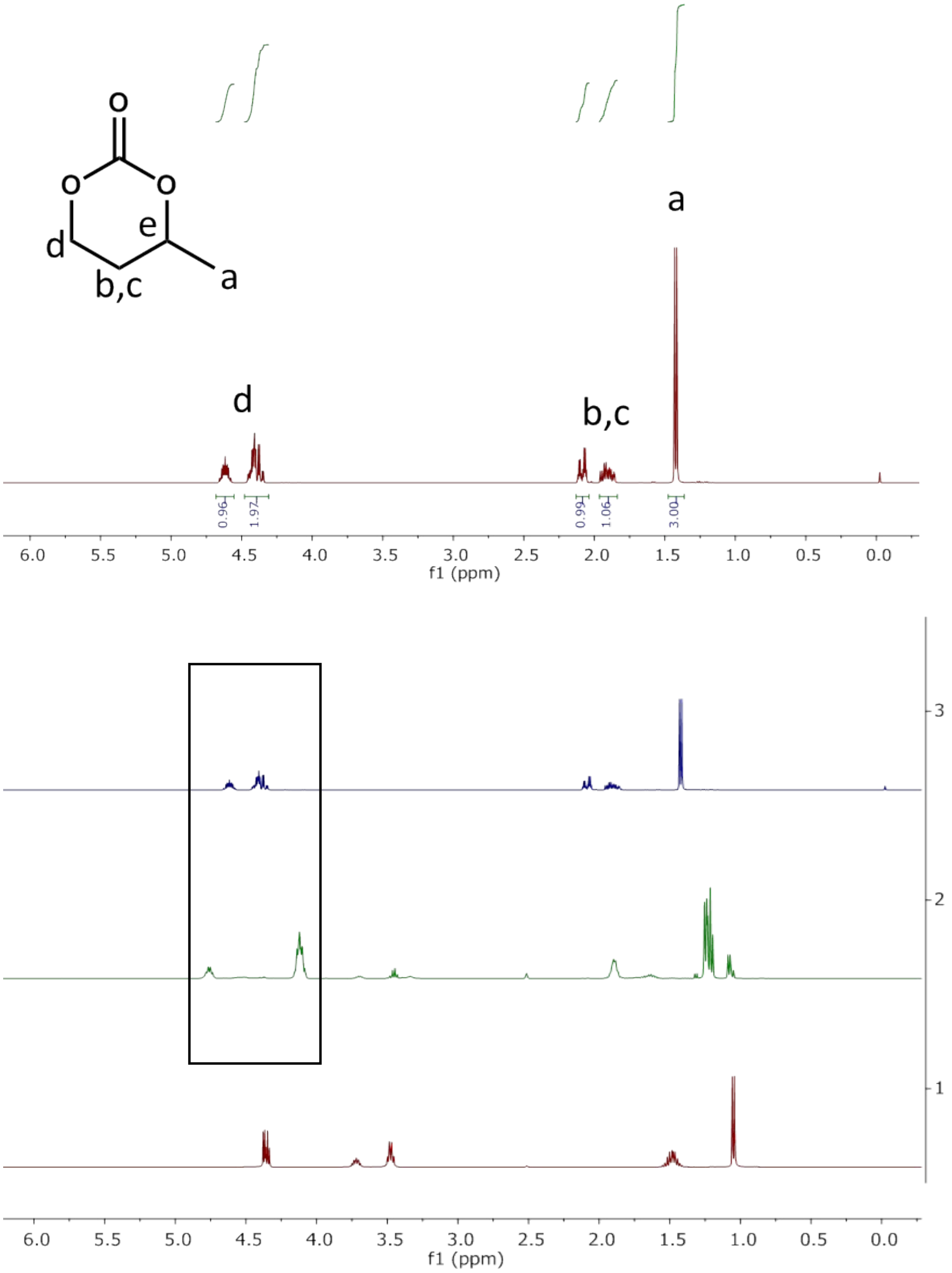

Figure S7 ^{1}H-NMR of 4-methyl-1,3-dioxan-2-one (6CC-5B).[5] Lower, ^{1}H-NMR compilation of 1) (±)-1,3-butanediol, 2) oligomers prior to cRCD and 3) 4-methyl-1,3-dioxan-2-one.

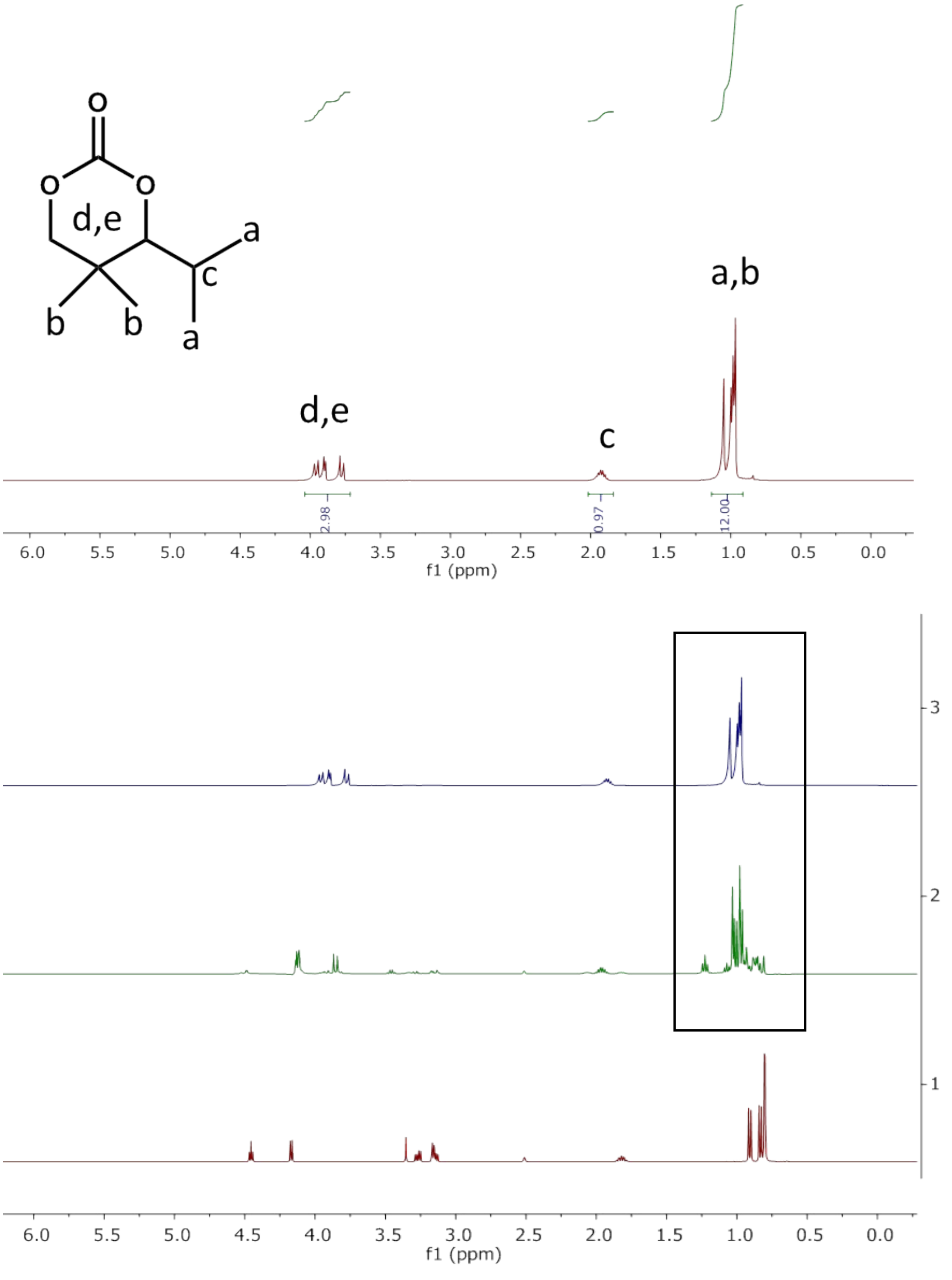

Figure S8 ^{1}H-NMR of 4-isopropyl-5,5-dimethyl-1,3-dioxan-2-one (6CC-7B).[6] Lower, ^{1}H-NMR compilation of 1) 2,2,4-trimethyl-1,3-pentanediol, 2) oligomers prior to cRCD and 3) 4-isopropyl-5,5-dimethyl-1,3-dioxan-2-one.

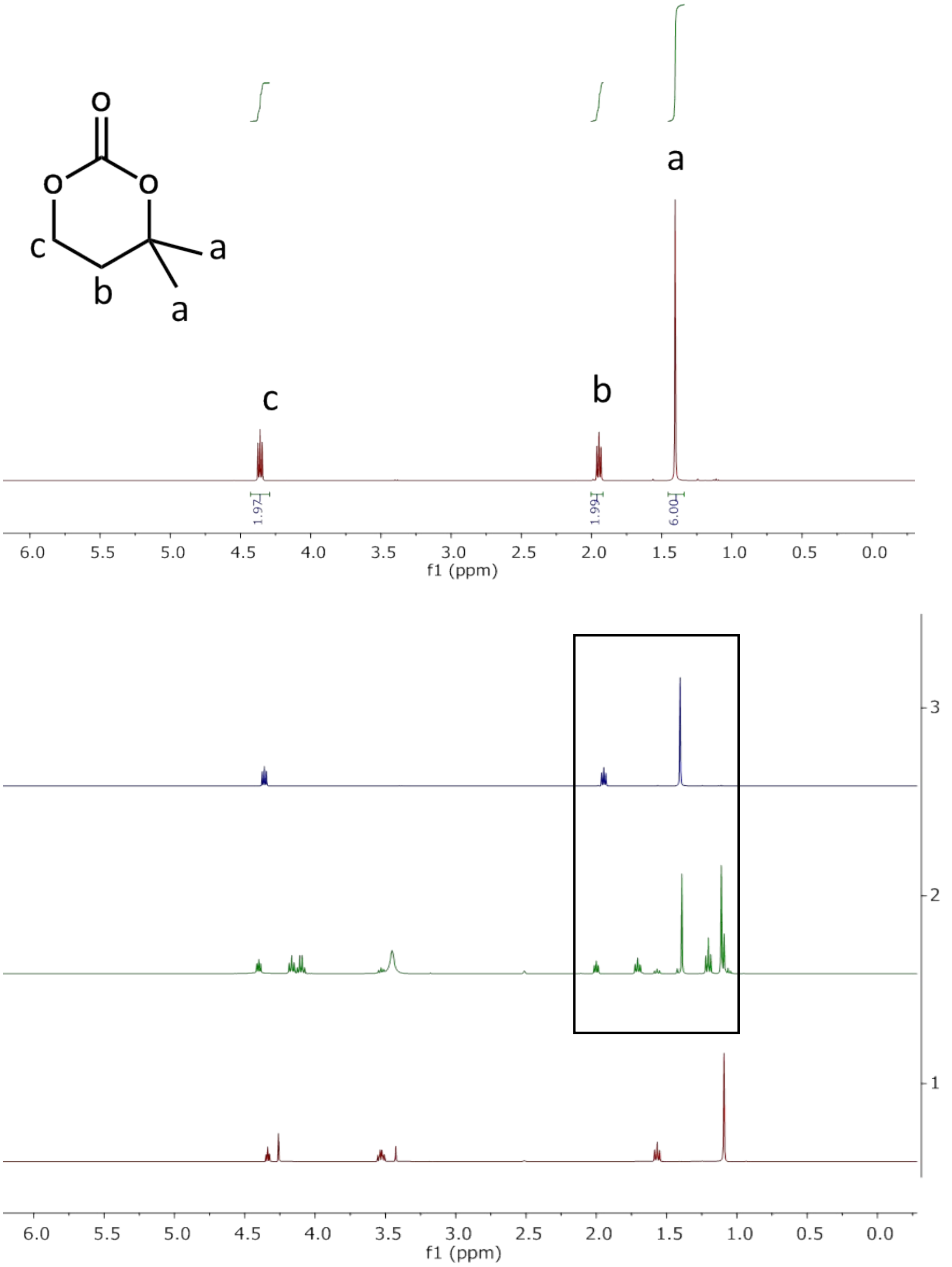

Figure S9 ^{1}H-NMR of 4,4-dimethyl-1,3-dioxan-2-one (6CC-6B).[3] Lower, ^{1}H-NMR compilation of 1) 3-methyl-1,3-butanediol, 2) oligomers prior to cRCD and 3) 4,4-dimethyl-1,3-dioxan-2-one.

References

1 Y. L. Hu and R. Xing, *Catal. Letters*, 2017, **147**, 1453–1463.

2 J. Rintjema, W. Guo, E. Martin, E. C. Escudero-Adán and A. W. Kleij, *Chem. - A Eur. J.*, 2015, **21**, 10754–10762.

3 S. H. Pyo and R. Hatti-Kaul, *Adv. Synth. Catal.*, 2016, **358**, 834–839.

4 T. F. Al-Azemi, H. H. Dib, N. A. Al-Awadi and O. M. E. El-Dusouqui, *Tetrahedron*, 2008, **64**, 4126–4134.

5 P. Brignou, J.-F. Carpentier and S. M. Guillaume, *Macromolecules*, 2011, **44**, 5127–5135.

6 Z.-H. Zhou, Q.-W. Song and L.-N. He, *ACS Omega*, 2017, **2**, 337–345.
